# Cellular activity upregulation of the thermolabile p53 cancer mutant Y220C by small molecule indazole derivatives

**DOI:** 10.1038/s41420-025-02781-6

**Published:** 2025-11-07

**Authors:** Raniya Khadiullina, Vitaly Chasov, Elvina Gilyazova, Damir Davletshin, Regina Mirgayazova, Rimma Mingaleeva, Joseph R. Stephenson Clarke, Matthias GJ Baud, Emil Bulatov

**Affiliations:** 1 Institute of Fundamental Medicine and Biology, Kazan Federal University, Kazan, Russia; 2 School of Chemistry and Chemical Engineering, University of Southampton, Southampton, UK; 3 Shemyakin-Ovchinnikov Institute of Bioorganic Chemistry, Russian Academy of Sciences, Moscow, Russia

**Keywords:** Screening, Tumour-suppressor proteins

## Abstract

The *TP53* gene is one of the most frequently mutated genes in human cancers. Mutations often result in loss of tumor-suppressive functions and acquisition of oncogenic properties by p53, contributing to tumor progression and resistance to therapy. Among structural p53 mutations, Y220C is particularly notable for creating a surface-exposed hydrophobic pocket that destabilizes the protein while preserving partial function, making it a promising target for pharmacological reactivation. In this study, we performed a structure-guided phenotypic screen of an in-house heterocyclic compound library to identify novel small-molecule modulators of p53-Y220C. This led to the identification of a series of (1H-pyrrol-1-yl)indazole derivatives (JC16, JC36, JC65), structurally inspired by known Y220C binders. JC16 and JC36 exhibited selective cytotoxicity and pro-apoptotic activity in p53-Y220C mutant cancer cell lines, with minimal effects in wild-type or p53-null cells. These compounds induced a mutant-to-wild-type conformational shift in cellular p53-Y220C, accompanied by transcriptional activation of canonical p53 target genes, including *BBC3 (PUMA)* and *MDM2*. Western blot analysis revealed that in HUH7 cells, these effects occurred without a corresponding increase in total p53 protein levels, suggesting a mechanism based on conformational reactivation. Our findings position JC16 and JC36 as early-stage chemical leads with potential to restore mutant p53 function in a context-dependent manner. While their exact mechanism of action remains to be fully elucidated, these results provide a foundation for further development of indazole-based scaffolds as reactivators of the p53-Y220C mutant in cancer therapy.

## Introduction

The p53 protein is a critical transcription factor that helps maintain genome integrity by activating downstream genes involved in cell cycle arrest, apoptosis, and DNA repair [1]. The *TP53* gene is one of the most frequently mutated genes in human cancers [2, 3]. The majority of oncogenic p53 mutations are missense mutations located in the DNA-binding domain (DBD) of the protein [4–7]. These mutations can be categorized into two types: “structural” mutations, which destabilize the tertiary structure of the p53 protein, and “contact” mutations, which disrupt crucial electrostatic interactions with target DNA sequences. Both mutation types lead to the loss of p53 transcriptional activity. In addition to this loss-of-function (LOF) effect, most mutant p53 proteins acquire oncogenic properties through a gain-of-function (GOF) mechanism [8]. Oncogenic mutant p53 can exert a dominant-negative effect on wild-type (WT) p53, partly through heteromerization between the mutant and WT forms, forming functionally inactive tetrameric complexes [9]. Furthermore, the mutant p53 protein promotes uncontrolled cell proliferation, inhibits apoptosis, confers resistance to antitumor agents, and facilitates invasion and metastasis [10, 11].

The Y220C mutation is one of the most prevalent missense mutations in the p53 protein, ranking as the ninth most common overall. It is found in over 125,000 newly diagnosed cancer cases globally each year [4, 7]. The mutation involves the substitution of the phenolic tyrosine side chain with a cysteine thiol (“big to small” mutation) at position 220, creating a narrow hydrophobic pocket on the surface of the DBD. This alteration reduces the protein’s thermodynamic stability and transcriptional activity [12]. Previous studies have shown that certain p53 mutants, including Y220C, can partially restore their tertiary structure and exhibit transcriptional activity similar to WT p53 at sub-physiological temperatures [13]. This finding supports the rationale for using small molecules to thermally and conformationally stabilize these partially denatured mutant proteins, thereby reactivating their transcriptional functions [14, 15]. Importantly, the pocket formed by the Y220C mutation is located away from the p53 protein active regions responsible for DNA binding and protein-protein interactions, presenting a unique therapeutic opportunity to specifically target this mutation without disrupting the protein’s essential functional domains. Thus, selective binding of a small chemical agent to this distinct pocket could stabilize the mutant p53 protein, preserving its ability to bind to native targets, and restore its functional activity without interfering with critical interactions [16].

Currently, several mechanistically distinct small-molecule drug candidates have been developed to target mutant p53 and treat malignancies. Notable examples include APR-246, COTI-2, and various 2-sulfonylpyrimidine derivatives, which have been explored as p53 cysteine capping agents (Michael acceptors) and Zinc(II) ion transporters [17–19]. These compounds suppress cancer cell proliferation presumably by reactivating mutant p53 and restoring, at least partially, its WT conformation [20, 21]. However, previous studies have also indicated that at least some of these reactive agents significantly increase intracellular levels of reactive oxygen species, suggesting that their effects may involve p53-independent and/or indirect mechanisms [22]. Additionally, we and colleagues have reported several in vitro, non-covalent p53-Y220C stabilizers utilizing iodophenol, carbazole, and pyrazole scaffolds, which offer alternative approaches for mutant p53 reactivation [23–25].

Eprenetapopt (APR-246), developed by Aprea Therapeutics, is a small-molecule Michael acceptor designed to reactivate mutant or inactivated p53 proteins by restoring their normal conformation and function, thereby inducing programmed cell death in cancer cells. Preclinical studies demonstrated its anti-tumor activity across a range of solid and hematological cancers, including myelodysplastic syndromes (MDS), acute myeloid leukemia (AML), and ovarian cancer. Eprenetapopt has shown strong synergy when combined with traditional chemotherapies and emerging anti-cancer therapies, such as immuno-oncology agents. A Phase 1/2 clinical trials have highlighted its favorable safety profile and promising clinical responses in *TP53*-mutated malignancies. However, while a Phase 3 trial for *TP53*-mutant MDS did not meet its primary endpoint, ongoing trials are investigating its efficacy in both hematological and solid tumors. Eprenetapopt has received multiple designations from the U.S. Food and Drug Administration (FDA), including Breakthrough Therapy, Orphan Drug, and Fast Track for MDS and AML, as well as Orphan Drug designation from the European Medicines Agency [26, 27].

More recently, PMV Pharma developed Rezatapopt (PC14586), a first-in-class, orally bioavailable small-molecule reactivator specifically targeting the p53-Y220C mutation. This compound stabilizes the thermally unstable mutant protein by binding to the unique pocket created by the mutation, restoring its wild-type conformation, reactivating its transcriptional activity, and promoting the expression of key tumor-suppressor genes [28, 29]. Rezatapopt has been granted FDA Fast Track designation for the treatment of patients with locally advanced or metastatic solid tumors harboring the *TP53* Y220C mutation and is currently undergoing evaluation in the tumor-agnostic PYNNACLE Phase 2 clinical trial (NCT04585750) to generate data supporting its regulatory approval.

In Phase 1 monotherapy, Rezatapopt demonstrated a favorable safety profile. Among patients treated with a daily dose of 2000 mg, an objective response rate (ORR) of 38% was observed, with a median duration of response of 7 months. The registrational Phase 2 study is currently evaluating Rezatapopt monotherapy in patients with *TP53* Y220C-mutated and KRAS wild-type advanced solid tumors, including ovarian, lung, breast, and endometrial cancers. Enrollment for this trial is ongoing, with interim data anticipated by mid-2025. Conversely, the Phase 1b combination therapy with pembrolizumab did not demonstrate a clinically meaningful benefit, leading to the discontinuation of this cohort.

A recent preclinical study also identified KG13, an azaindole derivative that covalently modifies the cysteine residues of p53-Y220C, reactivates target genes, and enhances the thermal stability of mutant p53 to WT levels [30]. Despite its promise, only a limited number of small molecules targeting mutant p53 have progressed to clinical trials, including APR-246, arsenic trioxide (ATO), COTI-2, and PC14586. More broadly, most molecules targeting mutant p53, whether at clinical or preclinical stages, face significant challenges. These include suboptimal binding affinity, intrinsic toxicity, indirect or incompletely understood mechanisms of action, and other difficulties that hinder comprehensive biological evaluation. These limitations underscore the importance of continued efforts to identify novel leads and pharmacophores capable of reactivating mutant p53 in cells. Discovering new scaffolds is not only essential for optimizing more effective reactivators of p53, especially p53-Y220C, but also holds the potential to deepen our understanding of p53 biology and its critical role in tumor suppression. Novel scaffolds that effectively reactivate mutant p53 could lead to therapies targeting specific mutations while simultaneously enhancing the overall functionality of the p53 pathway, paving the way for improved cancer treatment strategies.

**Fig. 1:**
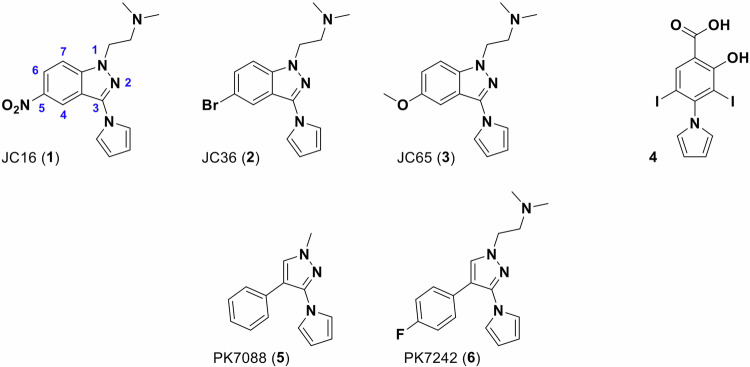
Chemical structures of the identified (1H-pyrrol-1-yl)indazole derivatives JC16 (**1**), JC36 (**2**), and JC65 (**3**), characterized by substitutions at the 5-position (NO_2_, Br, OCH_3_, respectively). Numbering of the indazole ring atoms is indicated in blue. The previously reported p53-Y220C ligand, (1H-pyrrol-1-yl)benzoic acid (**4**), is shown for comparison. PK7088 (**5**) and PK7242 (**6**) were previously identified as p53 reactivators by Liu and colleagues [25].

In this study, we applied cell-based phenotypic assays and biophysical methods to characterize three novel, structurally related compounds (JC16 (**1**), JC36 (**2**), and JC65 (**3**)) identified from a targeted similarity screen of an in-house heterocyclic library. These compounds share a common (1H-pyrrol-1-yl)indazole scaffold, featuring an ionizable *N*,*N*-dimethylethanamine moiety at position 1, a pyrrole ring at position 3, and distinct substituents at position 5 (nitro, bromo, or methoxy, respectively; Fig. 1). Our findings show that these compounds selectively reduce the viability of cancer cells harboring the p53-Y220C mutation and induce apoptosis in a mutation-dependent manner. Notably, JC16 and JC36 triggered a conformational shift in cellular p53-Y220C toward a wild-type-like state, which correlated with increased transcriptional activation of p53 target genes, supporting their potential as functional reactivators of mutant p53.

## Results and discussion

### Compounds library and selection

To identify novel chemical scaffolds capable of modulating the cellular activity of the thermolabile p53-Y220C mutant, we employed a targeted screening approach informed by previously validated small-molecule binders. Our strategy was motivated by prior work from Liu and colleagues, who reported the 3-(1H-pyrrol-1-yl)-1H-pyrazole derivative PK7088 (**5**, Fig. 1) as a small molecule capable of partially restoring wild-type-like conformation and transcriptional function to p53-Y220C in cancer cells [25]. While PK7088 (**5**) demonstrated modest thermal stabilization of the mutant p53 DNA-binding domain (ΔTm ≈ 1 °C) and relatively weak in vitro binding affinity (*K*_d_ ≈ 140–225 µM), it produced notable effects in cellular assays. Structural studies of the more soluble analog PK7242 (**6**, Fig. 1) confirmed specific engagement with the Y220C-induced surface pocket, providing strong structural validation of this site as a druggable target.

Building on these findings, we hypothesized that chemically related heterocyclic scaffolds might retain or improve upon the pocket-targeting properties of the pyrazole core while offering opportunities for increased cellular efficacy. To this end, we conducted a ligand-based similarity search across an in-house library of over 5,000 drug-like heterocyclic compounds maintained at the University of Southampton. This curated library consists primarily of unpublished and structurally diverse molecules, many of which feature privileged pharmacophores suitable for protein–ligand interactions.

Our screening pipeline consisted of two stages. First, compounds were filtered for structural similarity to the 3-(1H-pyrrol-1-yl)-1H-pyrazole moiety using substructure matching tools. Hits from this step were then evaluated based on key physicochemical properties associated with drug-likeness and cell permeability, including molecular weight ( > 250 Da), calculated lipophilicity (logP/D between 1 and 5), and predicted aqueous solubility ( > 100 µM). These parameters were selected to enrich for compounds with favorable absorption and bioavailability profiles, while ensuring compatibility with downstream biological assays. Property calculations were performed using SwissADME (http://www.swissadme.ch/), a widely used and validated tool for early-phase compound evaluation.

This process ultimately yielded a focused set of candidate molecules, among which the indazole-based derivatives JC16 (**1**), JC36 (**2**), and JC65 (**3**) (Fig. 1) were prioritized for further evaluation due to several converging factors: (i) their incorporation of a 5-substituted (1H-pyrrol-1-yl)indazole core, structurally analogous to the pharmacophore of PK7088 (**5**); (ii) favorable predicted physicochemical properties, including reasonable aqueous solubility ( > 100 µM) and balanced lipophilicity (logP between 2.3 and 3.5), consistent with drug-like behavior; and (iii) synthetic accessibility, which enabled the generation of sufficient material for parallel biological and biophysical characterization. Additionally, preliminary in silico modeling suggested that the electron-rich heterocyclic scaffold and substitution pattern may support hydrogen bonding and π–π stacking interactions compatible with the Y220C cavity environment. These combined factors made the compounds JC16 (**1**), JC36 (**2**), and JC65 (**3**) strong candidates for initial biological profiling and mechanistic evaluation.

This scaffold-focused approach, guided by validated precedent and supported by physicochemical profiling, allowed us to identify structurally novel indazole derivatives with potential to modulate p53-Y220C activity through mechanisms related to, but not necessarily limited to, pocket engagement.

#### (1H-pyrrol-1-yl)indazole derivatives induce stabilization of cellular p53-Y220C

One of the primary consequences of p53 mutations is protein unfolding, which disrupts its ability to bind DNA and activate the transcription of target genes [12, 31]. Correct folding is critical for p53 tumor-suppressive functions. In this study, we identified small-molecule (1H-pyrrol-1-yl)indazole derivatives **1**–**3** through a phenotypic screen of in-house heterocyclic compound libraries, specifically designed to identify compounds capable of upregulating p53 signaling in mutant p53 cell lines. Here, we evaluated whether compounds **1**–**3** could stabilize and re-activate the thermally unstable p53-Y220C mutant in cells. Our primary focus was to investigate the ability of JC16 (**1**) and JC36 (**2**) to induce a conformational shift in p53-Y220C, as measured by intracellular staining (*vide infra*).

**Fig. 2:**
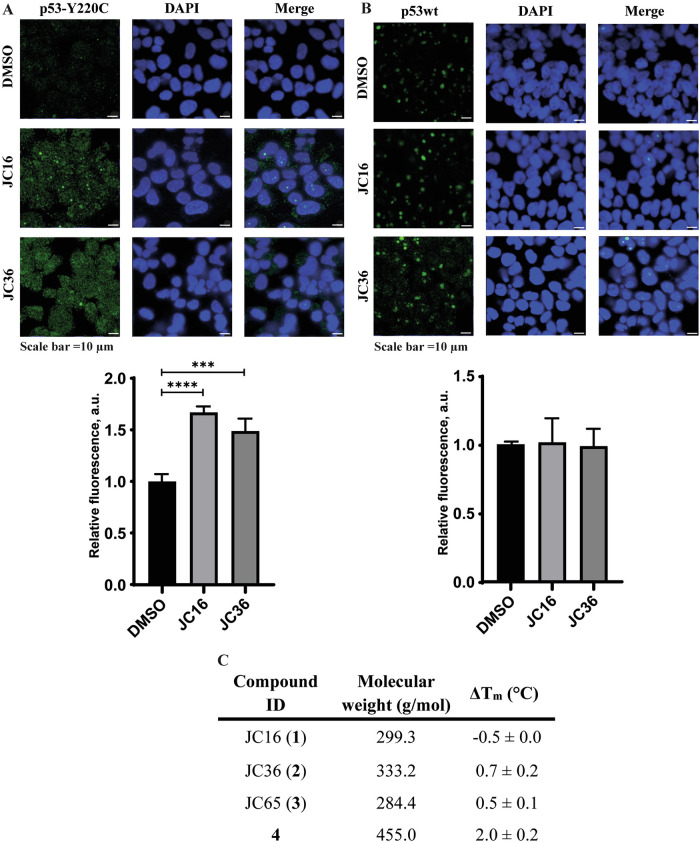
Induction of wild-type-like conformation and thermal stabilization of p53-Y220C by small molecule compounds. **A** Representative immunofluorescence images of HUH7 p53-Y220C cells treated with 60 µM JC16 (**1**), JC36 (**2**), or DMSO control, and stained with PAb1620 antibody, which recognizes the wild-type conformation of p53 (green). Nuclei were counterstained with DAPI (blue). **B** Parallel analysis in MCF7 p53 wild-type cells treated under the same conditions, showing no significant change in PAb1620 fluorescence. (Lower panels A and B): Quantification of fluorescence intensity was performed using ImageJ software (NIH, USA). Data are presented as mean ± SD (n = 3). Statistical significance was evaluated using one-way ANOVA with Tukey’s multiple comparison test (***p < 0.001; ****p < 0.0001). **C** Thermal stabilization (ΔTm) of purified p53-Y220C DBD upon binding of JC16, JC36, JC65, or iodophenol reference compound 4, as measured by differential scanning fluorimetry (DSF). Assays were conducted using 10 μM protein, 500 μM compound, and 10× SYPRO Orange dye. Values represent mean ± SD of three independent measurements.

To assess compound-mediated refolding of mutant p53-Y220C we used the PAb1620 antibody, which specifically recognizes the WT conformation of the p53 protein. This antibody binds a specific region on the DBD surface that is only exposed in the correctly folded WT p53 protein [32]. Treatment of HUH7 p53-Y220C cells with JC16 (1) or JC36 (2) led to a notable increase in fluorescence intensity, indicating a mutant-to-wild-type conformational change. This was confirmed by intracellular staining with the PAb1620 antibody (Fig. 2A). In contrast, treatment of MCF7 p53wt cells with JC16 (1) or JC36 (2) did not significantly alter the amount of WT p53 protein (Fig. 2B). These results suggest that JC16 (1) and JC36 (2) selectively induce a partial conformational shift in mutant p53-Y220C, promoting a wild-type-like status. These findings align with previous studies on mutant p53-reactivating compounds, such as COTI-2, APR-246, and PK11007, which similarly demonstrated increased stabilization and correct folding of mutant p53 protein in tumor cells [33–35]. This supports the therapeutic potential of (1H-pyrrol-1-yl)indazole derivatives as mutant p53-targeting agents that promote reactivation of its tumor-suppressive functions.

To assess conformational changes in the p53-Y220C protein, immunofluorescence analysis using the conformation-specific PAb1620 antibody was conducted exclusively in HUH7 cells, which endogenously express the p53-Y220C mutant. Although the MCF7 p53-Y220C cell line was used in other experiments within this study, it was not included in the PAb1620-based assay due to technical limitations. In preliminary tests, this model exhibited low and inconsistent fluorescence signal, likely reflecting reduced antibody accessibility or variable protein expression resulting from lentiviral reconstitution. In addition, Western blot analysis revealed that treatment with JC16 (**1**) and JC36 (**2**) in MCF7 p53-Y220C cells led to an increase in total p53 protein levels, which complicates the interpretation of any observed increase in PAb1620 signal, as it could be confounded by overall protein accumulation. For these reasons, we selected HUH7 as a more robust and reliable model to examine compound-induced stabilization and wild-type-like conformational shifts of the mutant p53 protein. This experimental choice is acknowledged as a limitation, and future studies will aim to validate these conformational effects across a broader panel of p53-Y220C models.

We evaluated the effect of compounds **1**–**3** on the thermostability of the p53-Y220C DBD using differential scanning fluorimetry (DSF). The melting temperature (T_m_) of p53-Y220C was assessed in the context of a stabilized quadruple mutant DBD (QM; M133L/V203A/N239Y/N268D). Indazole derivatives **1**–**3** had minimal impact on the T_m_ of p53-Y220C DBD, with thermal shifts of less than 1 °C at compound concentration of 500 µM (Figs. 2C and 1S). In contrast, the reference iodophenol derivative **4** produced a notable thermal shift of 2.0 °C under the same conditions, consistent with a measured *K*_d_ of 21 µM, as reported previously [36]. These in vitro binding studies suggest that compounds **1**–**3** exert their p53-Y220C re-folding and stabilization effects through an indirect mechanism, rather than by directly binding to the protein.

#### Effect of Compounds on Viability of Cell Lines with Different p53 Status

**Fig. 3:**
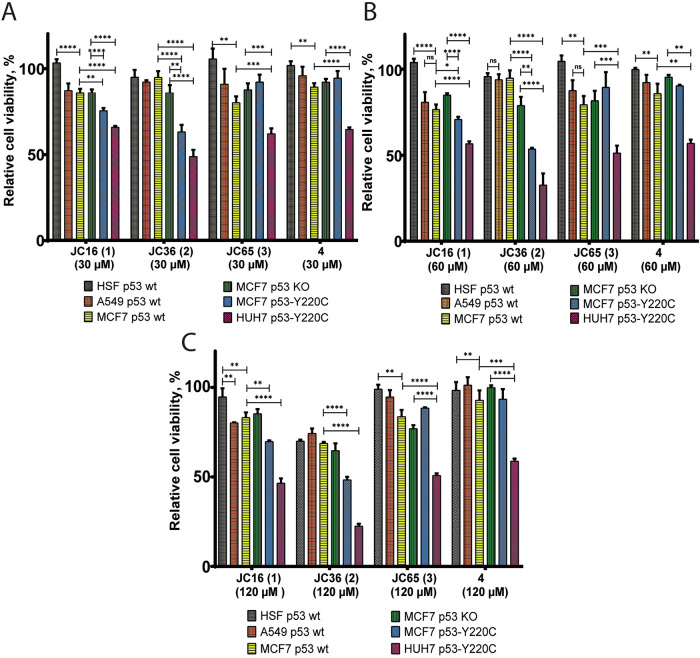
Dose-dependent effects of indazole derivatives on cell viability across cell lines with different p53 statuses. Cell viability was assessed using the MTS assay following 48-hour treatment with JC16 (**1**), JC36 (**2**), JC65 (**3**), and reference compound **4** at concentrations of 30 µM (**A**), 60 µM (**B**), and 120 µM (**C**). The panel includes cell lines representing various p53 statuses: HSF (non-cancerous, p53 wild-type), A549 and MCF7 (p53 wild-type), MCF7 p53 KO (p53-null), and MCF7 p53-Y220C and HUH7 p53-Y220C (mutant p53-Y220C). Viability was calculated relative to DMSO-treated controls (set at 100%), based on formazan absorbance at 490 nm. Data are presented as mean ± SEM from two biological replicates, each performed in triplicate. Statistical analysis was performed using one-way ANOVA with Tukey’s multiple comparison test (*p < 0.05; **p < 0.01; ***p < 0.001; ****p < 0.0001; ns = not significant).

We evaluated the viability of several cell lines with varying p53 statuses following treatment with compounds **1–3** and the control compound **4** (a known in vitro binder, *K*_d_ = 21 µM) for 48 h at concentrations of 2.5 µM, 5 µM, 10 µM, 20 µM, 30 µM, 60 µM, and 120 µM. The tested cell lines included WT p53 (MCF7, human breast adenocarcinoma; A549, human lung adenocarcinoma), p53-Y220C mutant (HUH7, human hepatocarcinoma; MCF7 p53-Y220C), p53 knockout (MCF7 p53 KO cell line generated via CRISPR/Cas9), and a non-cancerous fibroblast cell line (HSF). The MCF7 p53 KO line was generated using the CRISPR/Cas9 technique, and the MCF7 p53-Y220C line was subsequently created through lentiviral transduction of the p53-Y220C mutant into MCF7 p53 KO cells (see Materials and Methods). We hypothesized that the compounds would significantly reduce the viability of cancer cell lines harboring the p53-Y220C mutation while showing limited cytotoxicity in p53 wild-type or p53 knockout cell lines. Indeed, all compounds demonstrated a dose-dependent reduction in the viability of HUH7 p53-Y220C cells at concentrations above 30 μM while maintaining relatively low cytotoxicity against other cell lines within the same concentration range (Fig. 3). JC36 (**2**) displayed the highest cytotoxicity toward HUH7 p53-Y220C cells, reducing relative cell viability to 48%, 32%, and 22% at concentrations of 30 µM, 60 µM, and 120 µM, respectively (Fig. 3 and Table 1S). Similarly, in MCF7 p53-Y220C cells, JC36 **(2)** reduced cell viability to 63%, 53%, and 48% at 30 µM, 60 µM, and 120 µM, respectively. JC16 **(1)** also decreased the viability of MCF7 p53-Y220C cells but had minimal effects on wild-type p53 (MCF7 and A549) and knockout (MCF7 p53 KO) cell lines (Fig. 3 and Table 1S). In contrast, the non-cancerous HSF fibroblast cell line was the least affected by the tested compounds, maintaining viability close to control levels in most cases. This suggests that compounds **1**–**3** selectively target p53-Y220C mutant cancer cells, with minimal impact on non-cancerous or p53 wild-type cell lines.

#### Evaluation of compound cytotoxicity using xCELLigence RTCA biosensor analysis

**Fig. 4:**
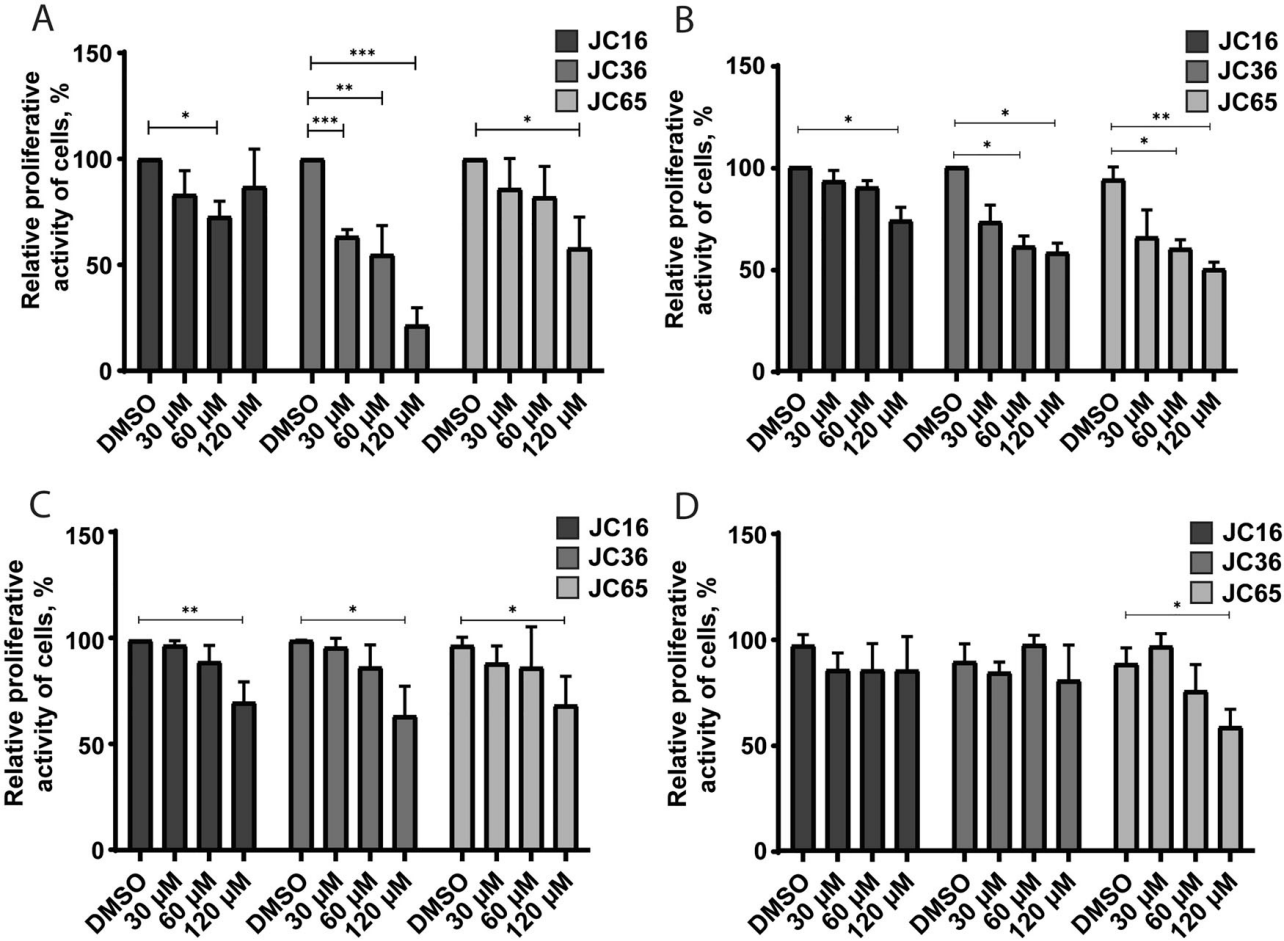
Real-time analysis of compound effects on cell proliferation across p53-defined cell lines. Proliferative activity of **A** HUH7 p53-Y220C, **B** MCF7 p53-Y220C, **C** MCF7 p53 wild-type, and **D** MCF7 p53 KO (p53-null) cells was evaluated using the xCELLigence real-time biosensor system following treatment with JC16 (**1**), JC36 (**2**), or JC65 (**3**). Cells were seeded on E-Plate 16 plates and treated after 24 h with compounds at concentrations of 30 µM, 60 µM, or 120 µM. Proliferation was monitored for 48 h post-treatment, and data were normalized to DMSO-treated control wells (set to 100%). Results are presented as mean ± SD of four technical replicates. Statistical significance was determined using one-way ANOVA followed by Tukey’s multiple comparison test (*p < 0.05; **p < 0.01; ***p < 0.001; ****p < 0.0001).

**Fig. 5:**
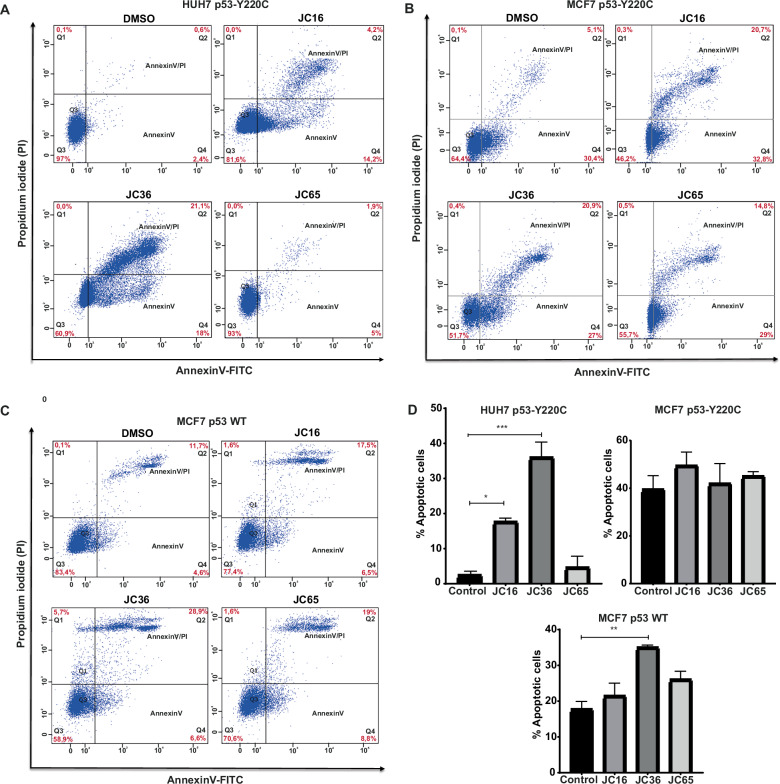
Flow cytometric assessment of apoptosis induced by indazole derivatives in cells with different p53 statuses. Annexin V-FITC/propidium iodide (PI) staining was used to assess apoptosis after 48-hour treatment with JC16 (**1**), JC36 (**2**), or JC65 (**3**) at 60 μM in **A** HUH7 p53-Y220C, **B** MCF7 p53-Y220C, and **C** MCF7 p53 wild-type cells. Flow cytometry dot plots show quadrant distribution: Q3—viable cells (Annexin V–/PI–), Q4—early apoptotic (Annexin V + /PI–), Q2—late apoptotic (Annexin V + /PI + ), and Q1—necrotic/dead cells (Annexin V–/PI + ). **D** Quantitative analysis of total apoptotic cells (Q2 + Q4) from each condition. Data are presented as mean ± SD (n = 3). Statistical significance was determined using one-way ANOVA followed by Tukey’s multiple comparison test (*p < 0.05; **p < 0.01; ***p < 0.001; ****p < 0.0001).

To further assess the cytotoxic effects of the compounds on tumor cells with varying p53 statuses, we utilized the xCELLigence RTCA real-time biosensor system. Cells were seeded onto specialized E-Plate 16 plates, and after a 24 h incubation period to allow adhesion, the compounds were added at concentrations of 30 μM, 60 μM, or 120 μM. Real-time plots of the cell index as a function of incubation time are presented in the Supplementary Data (Figs. 2S–5S).

In HUH7 p53-Y220C cells, treatment with JC36 (**2**) for 48 hours resulted in a substantial reduction in tumor cell proliferation by 37%, 46%, and 80% at concentrations of 30 μM, 60 μM, and 120 μM, respectively (Fig. 4A). In contrast, JC65 (**3**) exhibited reduced potency, significantly decreasing proliferation only at 120 μM, while JC36 **(2)** had negligible effects across all tested concentrations (Fig. 4A).

In MCF7 p53-Y220C cells, treatment with JC16 **(1)** at 120 μM for 48 hours reduced proliferation by 27% compared to DMSO-treated controls. Similarly, JC36 **(2)** and JC65 **(3)** reduced proliferation by 40% at 60 μM and by 42% and 50%, respectively, at 120 μM (Fig. 4B).

In MCF7 p53wt cells, treatment with JC16 **(1)**, JC36 **(2)**, and JC65 **(3)** at 120 μM for 48 hours resulted in decreased proliferation to 70%, 64%, and 69%, respectively (Fig. 4C). However, in MCF7 p53 KO cells, a significant reduction in proliferative activity was observed only after treatment with JC65 **(3)** at 120 μM for 48 hours (Fig. 4D).

Both the MTS assay and xCELLigence real-time cell analysis revealed consistent trends, demonstrating that the cytotoxicity of the compounds was most pronounced in tumor cell lines harboring the p53-Y220C mutation. Among the tested compounds, JC36 **(2)** exhibited the highest cytotoxicity against p53-Y220C mutant cells. Based on these cytotoxicity profiles, a working concentration of 60 μM was selected for subsequent experiments.

#### Effects of indazole derivatives on the induction of apoptosis

The pro-apoptotic activity of the indazole derivatives was assessed using annexin V-FITC/propidium iodide staining followed by flow cytometry in HUH7 p53-Y220C, MCF7 p53-Y220C, and MCF7 p53 wild-type cells (Fig. 5). Treatment with JC16 (1) and JC36 (2) significantly increased apoptosis in HUH7 cells, with JC16 inducing a ~ 7-fold and JC36 a ~ 14-fold increase in total apoptotic cells relative to DMSO-treated controls (Fig. 5A, D). These effects were primarily driven by increases in both early and late apoptotic cell populations. In MCF7 p53-Y220C cells, all three compounds (JC16, JC36, and JC65) induced elevated levels of apoptosis compared to the control, although the changes did not reach statistical significance (Fig. 5B, D), potentially due to cell line-specific differences in mutant p53 expression and signaling context. In MCF7 wild-type p53 cells, JC36 showed a modest but statistically significant increase in apoptosis ( ~ 1.9-fold), while JC16 and JC65 had less pronounced effects (Fig. 5C, D).

Taken together, these results support the notion that JC16 and JC36 promote apoptosis more effectively in cells harboring endogenous mutant p53-Y220C (HUH7) than in p53 wild-type or lentivirally transduced mutant models. This suggests a potential selective vulnerability of Y220C-mutant cancer cells to these compounds. Further investigation using a broader panel of isogenic p53 cell models and mechanistic apoptosis markers will be valuable to fully elucidate the pathways involved and to confirm the specificity of these compounds as mutant p53 reactivators.

#### Effect on transcriptional activity of mutant p53

**Fig. 6:**
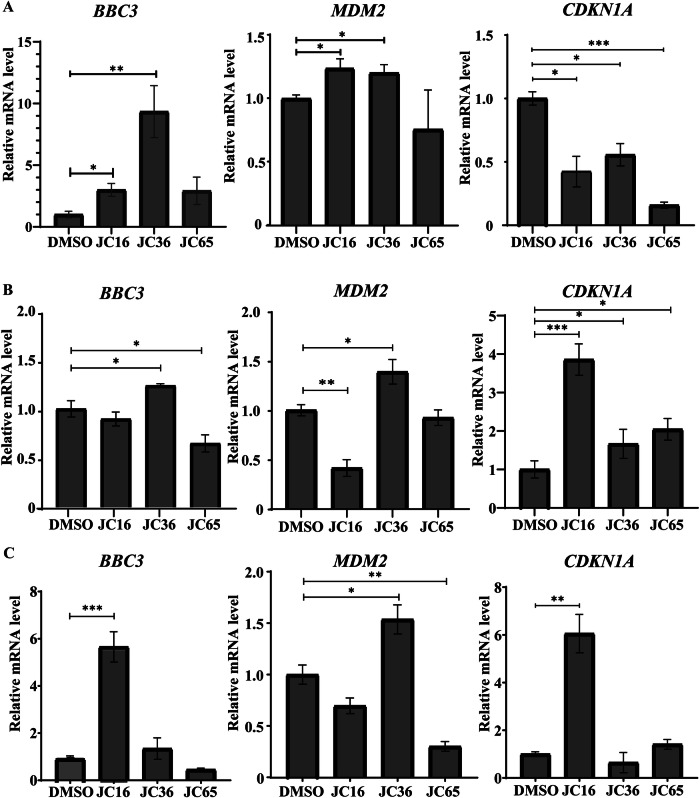
Modulation of p53 target gene expression by indazole derivatives in cells with different p53 statuses. Quantitative real-time PCR analysis of the mRNA expression levels of *BBC3 (PUMA)*, *MDM2*, and *CDKN1A (p21)* in **A** HUH7 p53-Y220C, **B** MCF7 p53-Y220C, and **C** MCF7 p53 wild-type cells following treatment with 60 µM of JC16 (**1**), JC36 (**2**), or JC65 (**3**) for 24 h. DMSO-treated cells (1%) were used as vehicle controls. Gene expression was normalized to *ACTB* and presented relative to the control group. Data are shown as mean ± SD (n = 3). Statistical significance was determined using one-way ANOVA followed by Tukey’s multiple comparison test (*p < 0.05; **p < 0.01; ***p < 0.001).

To determine whether the decreased viability of p53-Y220C cells treated with the compounds correlates with restored transcriptional activity of mutant p53, we measured the mRNA levels of key p53 target genes (*BBC3*, *CDKN1A (p21)*, and *MDM2*) using real-time PCR after 24 hours of incubation with 60 µM of compounds **1-3**. The p53 protein is known to influence both the extrinsic and mitochondrial pathways of apoptosis induction [37]. Additionally, p53 induces apoptosis by activating the transcription of several pro-apoptotic genes, including *BBC3* [38]. Treatment with JC36 **(2)** led to a significant increase in *BBC3* expression in both HUH7 p53-Y220C and MCF7 p53-Y220C cells (Fig. 6A, B), suggesting activation of the p53-dependent apoptotic pathway. In contrast, no significant changes in *BBC3* expression were observed in MCF7 p53wt cells following treatment with the same compound (Fig. 6C). Interestingly, JC16 **(1)** caused an increase in *BBC3* expression not only in HUH7 p53-Y220C cells but also in MCF7 p53wt cells. The literature suggests that, in some contexts, *BBC3* can also mediate p53-independent apoptosis [39]. This alternative pathway may involve factors such as reactive oxygen species (ROS), endoplasmic reticulum (ER) stress caused by ATP depletion, or calcium imbalance [40]. These findings highlight the potential of JC16 **(1)** to induce apoptosis through both p53-dependent and p53-independent mechanisms, warranting further investigation into its precise mode of action.

#### Regulation of *CDKN1A (p21)* and *MDM2* expression by compounds in cells with different p53 statuses

The p21 protein is a critical regulator of cell cycle checkpoints, inhibiting cell proliferation through direct interactions with CDKs and PCNA or indirectly at the transcriptional level [41]. Our analysis revealed that JC36 **(2)** and JC65 **(3)** increased *CDKN1A (p21)* expression in MCF7 p53-Y220C cells (Fig. 6B). Interestingly, JC16 **(1)** elevated *CDKN1A (p21)* expression not only in MCF7 p53-Y220C cells but also in MCF7 p53wt cells (Fig. 6B, C). However, none of the compounds induced *CDKN1A (p21)* expression in HUH7 p53-Y220C cells (Fig. 6A). This finding may reflect the involvement of p53-independent signaling pathways in regulating *CDKN1A (p21)* transcription. Indeed, *CDKN1A (p21)* expression can be modulated by various oncogenes, tumor suppressors, and inflammatory cytokines independent of p53 activity [42, 43].

We also observed an increase in *MDM2* mRNA levels in HUH7 p53(Y220C) cells following treatment with JC16 **(1)** (Fig. 6A). Notably, both mutant p53 (HUH7 p53-Y220, MCF7 p53-Y220C) and wild-type p53 (MCF7 p53wt) cells exhibited elevated *MDM2* expression after treatment with JC36 **(2)** (Fig. 6). This phenomenon can be attributed to the complex regulatory mechanisms governing p53-dependent transcription. The transcriptional activation of p53 target genes is not solely dependent on p53 stability and DNA-binding capability but is also influenced by post-translational modifications, interactions with cellular binding partners, and the specific sequence of p53 response elements [44]. Thus, the observed increase in *MDM2* expression in MCF7 p53wt cells highlights the intricate interplay of these regulatory mechanisms, which may differentially affect the transcriptional response to compounds in cells with distinct p53 statuses. These findings underscore the complexity of p53-mediated transcriptional regulation and the potential for context-specific effects of therapeutic compounds.

Although JC65 (**3**) was included in all phenotypic assays, its performance was consistently weaker compared to JC16 (**1**) and JC36 (**2**), particularly in cell viability, apoptosis, and gene expression assays. For this reason, we focused our mechanistic studies, such as p53 conformational analysis and transcriptional activation profiling, on the two more active compounds. JC65 (**3**) was retained in the analysis to support internal comparison within the chemical series, but the reduced magnitude of its biological effects limited its inclusion in detailed follow-up assays. This selective focus enabled clearer interpretation of mechanism while maintaining relevance to structure–activity relationships.

#### Effect of indazole derivatives on p53 protein expression

**Fig. 7:**
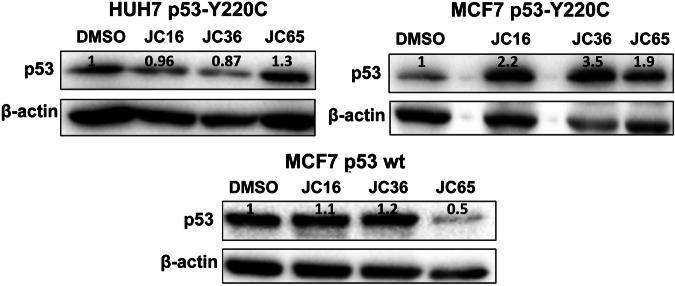
Effect of indazole derivatives on p53 protein expression in cell lines with distinct p53 statuses. Western blot analysis of p53 protein levels in **A** HUH7 p53-Y220C, **B** MCF7 p53-Y220C, and **C** MCF7 p53 wild-type cells following treatment with JC16 (**1**), JC36 (**2**), or JC65 (**3**) at 60 µM for 24 h. β-actin was used as a loading control. Relative p53 expression levels were quantified by densitometric analysis using ImageJ software and normalized to DMSO-treated controls (set to 1).

To determine whether the observed cellular effects of JC16 (**1**) and JC36 (**2**) were associated with changes in overall p53 protein levels, we performed Western blot analysis in HUH7 p53-Y220C, MCF7 p53-Y220C, and MCF7 p53 wild-type cells following compound treatment (Fig. 7). In HUH7 cells, which endogenously express mutant p53-Y220C, treatment with JC16 (**1**) or JC36 (**2**) did not lead to significant changes in total p53 protein levels, suggesting that the compounds do not broadly affect protein abundance in this model. A similar result was observed in MCF7 p53 wild-type cells, where p53 expression remained relatively stable across treatment conditions.

In contrast, MCF7 p53-Y220C cells exhibited a marked increase in p53 protein levels following treatment with JC16 (**1**) and JC36 (**2**). This difference may reflect cell line-specific responses related to the lentiviral reconstitution of mutant p53 in a knockout background, and may also indicate partial stabilization of the mutant protein under treatment. However, the absence of significant p53 upregulation in HUH7 cells, where the most robust pro-apoptotic and transcriptional effects were observed, suggests that the biological activity of JC16 (**1**) and JC36 (**2**) may be mediated more by conformational reactivation of mutant p53 rather than through increased expression alone.

Combined analysis of the qPCR and Western blot data provides mechanistic insight into how JC16 (**1**) and JC36 (**2**) modulate p53 activity across distinct cellular contexts. In HUH7 cells, which endogenously express the thermolabile p53-Y220C mutant, JC36 (**2**) treatment led to a significant increase in *BBC3 (PUMA)* and *MDM2* mRNA levels without a corresponding rise in total p53 protein, suggesting that transcriptional activation results from conformational reactivation rather than increased protein expression. JC16 (**1**) produced a similar, albeit less pronounced, effect. These findings support the hypothesis that both compounds can restore wild-type-like transcriptional function to mutant p53-Y220C in a protein-level-independent manner.

In contrast, in the MCF7 p53-Y220C model, which expresses mutant p53 via lentiviral transduction, treatment with JC16 (**1**) and JC36 (**2**) resulted in increased p53 protein levels alongside enhanced expression of *BBC3 (PUMA)*, *CDKN1A (p21)*, and *MDM2*. This pattern suggests that in this model, transcriptional reactivation may involve both conformational rescue and compound-induced protein stabilization. However, the lack of strong transcriptional responses in MCF7 p53 wild-type cells, particularly for *BBC3 (PUMA)*, and the absence of p53 upregulation in HUH7 cells argue against a purely expression-driven mechanism.

These results highlight the context-dependent nature of p53 target gene regulation and suggest that the transcriptional effects of JC16 (**1**) and JC36 (**2**) are not uniform across cell types. For example, the differential induction of *CDKN1A (p21)* and *BBC3 (PUMA)* across models points to gene-specific and chromatin-dependent regulatory dynamics. The robust activation of *BBC3 (PUMA)* and *MDM2* in HUH7 cells without p53 accumulation further supports a mechanism rooted in conformational restoration of mutant p53 activity.

Altogether, our findings indicate that JC16 (**1**) and JC36 (**2**) enhance the transcriptional activity of p53-Y220C through mechanisms that differ by cellular context, relying on direct reactivation in some models and stabilization in others. The observed transcriptional effects in MCF7 wild-type cells, particularly with JC16 (**1**), also raise the possibility of partial p53-independent activity or engagement with alternative stress pathways.

## Conclusions

The tumor suppressor p53 plays a central role in maintaining genomic integrity by regulating cell cycle checkpoints, DNA repair, and apoptosis. Mutations in *TP53* are found in approximately half of all human cancers, with the Y220C substitution being one of the most common structural mutations. This mutation introduces a surface-accessible hydrophobic pocket that destabilizes the p53 DNA-binding domain (DBD), making it an attractive target for small-molecule stabilization and reactivation strategies.

In this study, we investigated a series of (1H-pyrrol-1-yl)indazole derivatives, JC16 (**1**), JC36 (**2**), and JC65 (**3**), as candidate small-molecule reactivators of p53-Y220C. JC16 and JC36 exhibited selective anti-proliferative and pro-apoptotic activity in p53-Y220C mutant cancer cell lines, with minimal cytotoxicity in wild-type or p53-null backgrounds. Notably, these compounds induced a mutant-to-wild-type conformational shift in p53-Y220C, accompanied by increased transcriptional activity and upregulation of canonical p53 target genes such as *BBC3 (PUMA)*.

Although these compounds did not strongly stabilize the recombinant p53-Y220C DBD in vitro, their cellular activity suggests a mechanism of action that may involve indirect conformational reactivation or context-dependent stabilization within the cellular environment. This observation is consistent with previous reports on PK7088 (**5**) and PK7242 (**6**), which similarly enhanced the functional conformation of p53-Y220C without robust in vitro binding [25]. Whether JC16 and JC36 share this mechanism remains to be determined.

As early-stage leads identified through phenotypic screening, the precise molecular targets and mechanisms of JC16 (**1**) and JC36 (**2**) have yet to be fully elucidated. It is plausible that their effects involve more than one mode of action, potentially combining direct engagement with mutant p53 and indirect modulation of cellular stress or signaling pathways. Future studies employing biophysical target engagement assays (e.g., CETSA), transcriptomic analysis, and detailed structure–activity relationship investigations will be critical to validate their specificity and clarify their mechanistic underpinnings.

Furthermore, evaluating these compounds across a broader panel of p53-Y220C mutant models, including in vivo systems, will be essential to assess their therapeutic relevance and translational potential. Understanding stabilization mechanisms beyond direct pharmacological chaperoning may offer new avenues for treating p53-mutant cancers and complement existing reactivation strategies.

In summary, our findings highlight the potential of (1H-pyrrol-1-yl)indazole derivatives as structurally novel chemical scaffolds for the reactivation of mutant p53-Y220C. These results provide a compelling rationale for further development and mechanistic exploration of this compound class as prospective antitumor agents.

## Materials and methods

### Biophysics—protein expression and purification

The stabilized form of the p53-Y220C DBD (amino acids 94–312; quadruple mutant M133L/V203A/N239Y/N268D) was expressed and purified as previously described [45, 46]. Briefly, p53-Y220C DBD was produced by overexpression in *E. coli* BL21 (DE3) pLysS cells cultured in TB medium at 20 °C for 16 h. For initial purification, recombinant proteins were captured using Ni Sepharose 6 FastFlow affinity resin (GE Healthcare, Chicago, IL, USA) following the manufacturer’s instructions. Subsequently, hexahistidine tags were cleaved using TEV protease, with incubation conducted at 4 °C for 12 h in the presence of 0.5 mM EDTA, using a protease-to-protein ratio of 1:100 (w/w). Further purification was performed using a HiTrap Heparin HP column (GE Healthcare, Chicago, IL, USA) in 10 mM sodium phosphate buffer (pH 7.4), containing 5 mM DTT, with a NaCl gradient (0–1 M) for protein elution. Final purification was achieved via gel filtration chromatography using an ENrich™ SEC 70 column (Bio-Rad, Hercules, CA, USA) in a buffer comprising 20 mM Tris-HCl (pH 7.4), 150 mM NaCl, and 5 mM DTT. The purified proteins were concentrated using Vivaspin concentrators with a molecular weight cutoff of 10 kDa. Protein molecular weight and purity were confirmed by SDS-PAGE. Final protein samples were flash-frozen in liquid nitrogen and stored at −80 °C for future use.

### Biophysics—differential scanning fluorimetry (DSF)

The melting temperature (T_m_) of the p53 mutant was determined using differential scanning fluorimetry (DSF). DSF measurements were conducted on a LightCycler 480 thermocycler (Roche, Germany) with 10 μM protein (stabilized p53-Y220C DBD) and 10× SYPRO Orange dye (Sigma-Aldrich, USA) in an assay buffer containing 20 mM Tris-HCl (pH 7.4) and 150 mM NaCl. During thermal denaturation, the hydrophobic regions of the protein are exposed and interact with the hydrophobic dye, which then fluoresces. The fluorescence intensity is directly correlated with changes in protein folding (melting) [47]. To assess the effects of compounds on the protein melting temperature Tm (ΔT_m_), the assay buffer was supplemented with 1% DMSO (v/v) and either 500 μM ligand or no ligand (control). The Tm in the presence of compounds was determined from the extremum of the first derivative of the melting curve. ΔT_m_ values were calculated as ΔT_m_ = T_m_(protein + compound) – T_m_(protein alone). All measurements were performed in triplicate.

### Biological evaluation and cell culture

Human breast adenocarcinoma MCF7 (wild-type p53) and human lung carcinoma A549 (wild-type p53) cell lines were obtained from the American Type Culture Collection (ATCC). The human hepatoma Huh-7 (p53-Y220C) cell line was kindly provided by Prof. N. Barlev (Institute of Cytology, Russian Academy of Sciences, Russia). The human skin fibroblast (HSF) cell line was generously provided by Prof. A. Rizvanov (Kazan Federal University, Russia), with HSFs cultured as previously described [48]. The p53 knock-out (p53 KO) MCF7 cell line was derived from the MCF7 cell line using CRISPR/Cas9 gene editing [49]. The p53-Y220C MCF7 cell line was generated from the MCF7 (p53 KO) cell line using lentiviral transduction (described below). All cells were cultured in RPMI-1640 medium (PanEco, Russia) supplemented with 10% fetal bovine serum (FBS) (Hyclone, USA), 1 mM L-glutamine (PanEco, Russia), and a mixture of antibiotics (penicillin 5,000 U/ml and streptomycin 5,000 µg/ml) (PanEco, Russia) at 37 °C in a humidified atmosphere with 5% CO_2_.

### Biological evaluation—generation of the MCF7 p53-Y220C cell line via lentiviral transduction

To generate the expression constructs, the cDNA of the *TP53* gene was cloned into the pDONR221 plasmid vector using Gateway cloning technology (Invitrogen, USA). The cloning process involved a two-step PCR amplification of the *TP53* gene fragment. In the first step, gene-specific primers TP53-attB1 and TP53-attB2 (Table 1) were used to attach attB recombination sites. In the second step, adapter primers Adap-attB1 and Adap-attB2 (Evrogen, Russia) (Table 1) were used to finalize the PCR amplification and prepare the fragment for recombination. The amplified PCR fragments were subsequently recombined with the pDONR221 vector using BP Clonase enzyme mix, following the manufacturer’s protocol (Invitrogen, USA). The resulting clones were screened, and successful constructs were verified through Sanger sequencing to confirm the integrity and accuracy of the *TP53* gene insertion.

**Table 1:** Primer sequences for PCR amplification in Gateway cloning.

Primer name	Primer sequence
TP53–attB1	5’-AAAAGCAGGCTTCACCATGGAGGAGCCGCAGTCAGATCCTAGC-3’
TP53–attB2	5’-AGAAAGCTGGGTGTTAGTCTGAGTCAGGCCCTTCTGTCTT-3’
Adap-attB1	5’- GGGGACAAGTTTGTACAAAAAAGCAGGCT-3’
Adap-attB2	5’- GGGGACAAGTTTGTACAAAAAAGCAGGCT-3’

### Generation of lentiviral plasmid encoding p53-Y220C and transduction of MCF7 cells

The lentiviral plasmid pLX330-р53-Y220C, encoding the р53-Y220C protein gene, was constructed by sub-cloning the *TP53* gene from the donor vector (pDONR22-TP53) into the lentiviral plasmid pLX303 (#25897, Addgene, USA) via LR recombination using the Gateway™ LR Clonase™ II Enzyme mix (Invitrogen, USA) following the manufacturer’s protocol. To produce second-generation replication-incompetent lentiviruses (LVs), HEK293FT cells were transfected using calcium phosphate with three plasmids: the expression plasmid pLX330-р53-Y220C or pWPT-GFP (#12255, Addgene, USA), the psPAX2 packaging plasmid (#12260, Addgene, USA), and the pCMV-VSV-G envelope plasmid (#8454, Addgene, USA). Lentiviral particles (LV-p53-Y220C) were concentrated by ultracentrifugation at 26,000 rpm for 2 hours at 4 °C. Subsequently, the LV-p53-Y220C lentivirus was added to MCF7 p53 KO cells and incubated for 24 hours. Transduced cells were selected by culturing in medium supplemented with 8 μg/ml blasticidin (Thermo Fisher, USA) for two weeks. The successful incorporation of the Y220C mutation into the MCF7 cells was confirmed by Sanger sequencing.

### Treatment of cells with compounds

Stock solutions of the compounds were prepared by dissolving them in DMSO to a concentration of 20 mM. Prior to treatment, compounds were diluted to the desired concentrations while maintaining a constant final DMSO concentration of 1% across all samples. Vehicle control samples containing 1% DMSO without the compounds were included in each experiment.

### Cell viability assays

Cells were seeded into 96-well culture plates at a density of 5000 cells per well and allowed to adhere for 24 h. Following this incubation, cells were treated with the compounds at final concentrations of 2.5 μM, 5 μM, 10 μM, 20 μM, 30 μM, 60 μM, and 120 μM. After 48 hours of compound exposure, cell viability was assessed using an MTS assay. A solution containing 2 mg/ml MTS reagent (Promega, USA) and 150 μM phenazine methosulfate (Dia-M, Russia) was added to each well, and the plates were incubated at 37 °C in a humidified atmosphere with 5% CO_2_ for 3 h. The optical density was measured at 490 nm using an Infinite M200 microplate analyzer (Tecan, Switzerland). Each experiment was performed in triplicate, and cell viability was calculated as a percentage relative to the values in the vehicle control wells, which were set as 100%.

### Real-time cell proliferation analysis

Real-time cell analysis (RTCA) was conducted using the xCELLigence biosensor system (ACEA Biosciences, USA), a multiparameter cell culture assay platform designed to monitor cell viability and proliferation in real time under various treatment conditions. The system operates by continuously measuring electrical impedance across electrodes integrated into the wells of specialized plates, providing a cell index value that reflects the number of viable cells in the sample [50]. For the assay, 5 × 10^3^ cells were seeded into each well of an E-Plate 16 (ACEA Biosciences, USA) and incubated for 24 h to allow cell adhesion. Following this, compounds were added at final concentrations of 30 μM, 60 μM, and 120 μM and the cells were incubated for 72 h. The system recorded the cell index every 15 minutes throughout the experiment, with the recorded values corresponding to changes in electrical impedance, which are directly proportional to the number of living cells.

### Immunofluorescence staining

Cells were seeded into 6-well glass-bottom culture plates at a density of 2 × 10^5^ cells per well and allowed to adhere overnight. The following day, cells were treated with compounds at a concentration of 60 μM and incubated for 24 h. After treatment, cells were fixed with 4% paraformaldehyde and permeabilized using phosphate-buffered saline (PBS) containing 0.1% Triton X-100. Cells were incubated with primary antibodies against p53 (PAb1620, Merck, Germany) for 12 h at 4 °C. Subsequently, the cells were stained with a secondary antibody labeled with Alexa Fluor 647 A32728 (Merck, Germany) at a 1:1000 dilution. Nuclei were counterstained with DAPI (Fisher Scientific, USA). Fluorescence images were captured using an LSM 780 laser scanning confocal microscope (Carl Zeiss, Germany). Fluorescence intensity was quantified using ImageJ software (NIH, USA) to evaluate p53 expression levels.

### Apoptosis assay

Cells were seeded into 6-well culture plates at a density of 3 × 10^5^ cells per well and incubated overnight to allow attachment. The following day, cells were treated with compounds at a final concentration of 60 μM and cultured for 48 h. Apoptotic cell death was assessed using an apoptosis detection kit (BioLegend, USA) containing annexin V-FITC and propidium iodide (PI), following the manufacturer’s protocol. After staining, apoptosis levels were analyzed using a FACSAria III flow cytometer (BD Biosciences, USA).

### Western blotting

Cells were seeded into 6-well culture plates at a density of 3 × 10^5^ cells per well. The following day, the cells were treated with compounds at a final concentration of 60 μM and cultured for 24 hours. After treatment, the cells were collected and lysed in RIPA buffer (Thermo Fisher Scientific, USA), supplemented with 1 μl/ml of the “Protease and Phosphatase Inhibitor Cocktail with EDTA” (Thermo Fisher Scientific, USA). The concentration of total protein in the resulting lysates was determined using the Pierce BCA Protein Assay Kit (Thermo Fisher Scientific, USA). Protein lysates were then subjected to Tris-glycine sodium dodecyl sulfate-polyacrylamide gel electrophoresis (10% PAAG). Proteins were transferred from PAAG to PVDF membrane (Bio-Rad, USA) using the Trans-Blot SD Semi-Dry Transfer Cell blotting system (Bio-Rad, USA). Primary antibodies against p53 (Abcam, UK; ab1101) and β-actin (GenScript, USA; A00730) were used for detection. The membrane was developed with Clarity Western ECL Substrate (Bio-Rad, USA), and the chemiluminescent signal was captured using the ChemiDoc XRS Plus documentation system (Bio-Rad, USA). Band intensity was quantified densitometrically using ImageJ software (National Institutes of Health, USA).

### Quantitative polymerase chain reaction (qPCR)

Cells were seeded into 6-well culture plates at a density of 3 × 10^5^ cells per well and incubated overnight. The following day, cells were treated with compounds at a final concentration of 60 μM and cultured for 24 hours. Total RNA was isolated using TRIzol Reagent (Thermo Fisher Scientific, USA) according to the standard protocol. Reverse transcription was performed using the MMLV RT kit (Evrogen, Russia) to synthesize complementary DNA (cDNA). Quantitative real-time PCR was conducted using the 5x qPCRmix-HS premix (Evrogen, Russia). Primers were designed using the Primer-BLAST online tool (NCBI) and synthesized by Evrogen (Moscow, Russia) (Table 2). Relative mRNA expression levels were calculated using the ΔΔCt method, with beta-actin *(ACTB)* serving as the housekeeping reference gene. Each reaction was performed in triplicate to ensure accuracy.

**Table 2:** Primer and probe sequences for qPCR analysis.

Gene	Primer sequence
*MDM2*	Fwd: 5’TGTGCAAAGAAGCTAAAGAAAAGG Rev: 5’ AGGTTGTCTAAATTCCTAGGGTTAT Probe: 5’[HEX]ATTGGTTGTCTACATACTGGGCAGGG[BHQ2]
*BBC3 (PUMA)*	Fwd: 5’ GGGCCCGTGAAGAGCAAATG Rev: 5’ CTGGCTCAGGGAAGATGGCT Probe:5’[FAM]CGGTTGCTCCAGCCCGGCGC[BHQ1]
*CDKN1A (p21)*	Fwd: 5’ GCCTCCTCATCCCGTGTTCT Rev: 5’ GTACCACCCAGCGGACAAGT Probe: 5’ [HEX]AGCCGGCCCACCCAACCTCCG[BHQ2]
*ACTB*	Fwd: 5’ GCGAGAAGATGACCAGGATC Rev: 5’CCAGTGGTACGGCCAGAGG Probe: 5’[HEX]CCAGCCATGTACGTTGCTATCCAGGC[BH2]

### Statistical analysis

Statistical analyses were performed using GraphPad Prism version 9.3.1 (GraphPad Software, USA). Data are presented as mean ± standard deviation, with all experiments conducted in triplicate. A one-way analysis of variance (ANOVA) followed by Tukey’s multiple comparison test was used to evaluate the significance of differences between control and treated groups. Results were considered statistically significant at p < 0.05. Statistical significance levels are denoted as *p < 0.05, **p < 0.01, ***p < 0.001, and ****p < 0.0001.

## Supplementary information

Original Data_Western Blots (uncropped)

Supplementary data

## Data Availability

All data supporting the findings of this study are available from the corresponding author upon reasonable request. Full-length, uncropped images of the original Western blots are provided in the Supplementary Material.
