# Supplementary material for: Cellular activity upregulation of the thermolabile p53 cancer mutant Y220C by small molecule indazole derivatives
Source: Cell Death Discov. 2025 Nov 7;11:508. doi: 10.1038/s41420-025-02781-6 (PMC12594860; doi:10.1038/s41420-025-02781-6)
Supplement: Supplementary file 1 — Original Data_Western Blots (uncropped) [file 41420_2025_2781_MOESM1_ESM.docx]

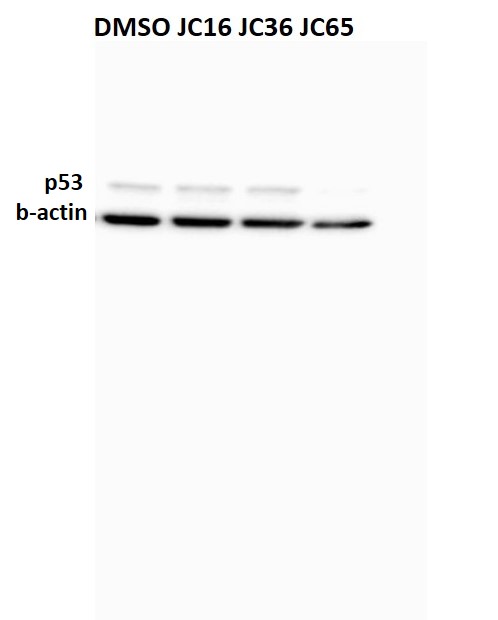

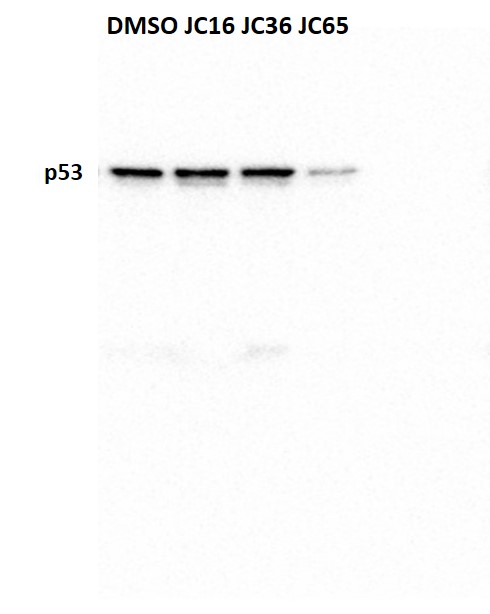


**Supplement for Figure 7. Effect of Compounds on the p53 Expression Levels in Cells with Different p53 Statuses.** Immunoblotting to determine the protein levels of p53 after JC16 (**1**), JC36 (**2**), and JC65(**3**) treatment for 24 h in MCF7 p53wt. Actin was used as a protein loading control. Left**:** shorter exposure time for p53; right: longer exposure time for p53


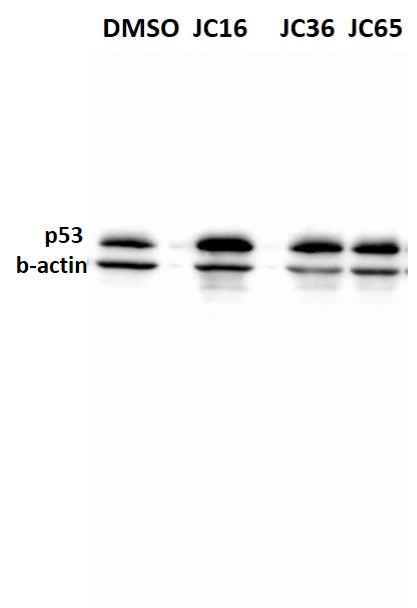


**Supplement for Figure 7. Effect of Compounds on the p53 Expression Levels in Cells with Different p53 Statuses.** Immunoblotting to determine the protein levels of p53 after JC16 (**1**), JC36 (**2**), and JC65(**3**) treatment for 24 h in MCF7 p53 Y220C. Actin was used as a protein loading control.

**
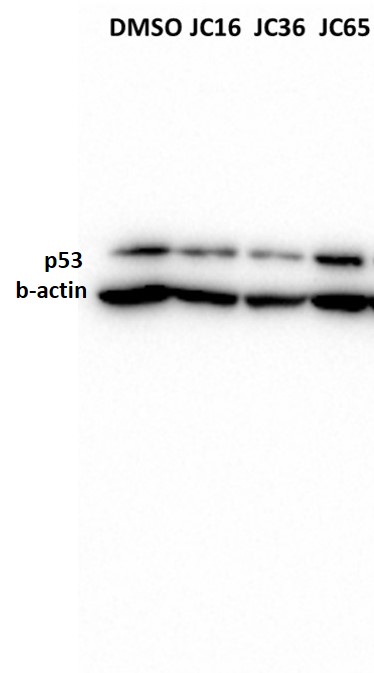
**

**Supplement for Figure 7. Effect of Compounds on the p53 Expression Levels in Cells with Different p53 Statuses.** Immunoblotting to determine the protein levels of p53 after JC16 (**1**), JC36 (**2**), and JC65(**3**) treatment for 24 h in HUH7 p53 Y220C. Actin was used as a protein loading control.
