# Supplementary material for: Cellular activity upregulation of the thermolabile p53 cancer mutant Y220C by small molecule indazole derivatives
Source: Cell Death Discov. 2025 Nov 7;11:508. doi: 10.1038/s41420-025-02781-6 (PMC12594860; doi:10.1038/s41420-025-02781-6)
Supplement: Supplementary file 2 — Supplementary data [file 41420_2025_2781_MOESM2_ESM.docx]

**Appendix. Supplementary data**

**Cellular Activity Upregulation of the Thermolabile p53 Cancer Mutant Y220C by Small Molecule Indazole Derivatives**

Raniya Khadiullina^1^, Vitaly Chasov^1^, Elvina Gilyazova^1^, Damir Davletshin^1^, Regina Mirgayazova^1^, Rimma Mingaleeva^1^, Joseph R. Stephenson Clarke^2^,
Matthias GJ Baud^2^ and Emil Bulatov^1,3^

*1 Institute of Fundamental Medicine and Biology, Kazan Federal University, Kazan 420008, Russia;*

*2 School of Chemistry, University of Southampton, Southampton SO17 1BJ, United Kingdom;*

*3 Shemyakin-Ovchinnikov Institute of Bioorganic Chemistry, Russian Academy of Sciences, 117997 Moscow, Russia*


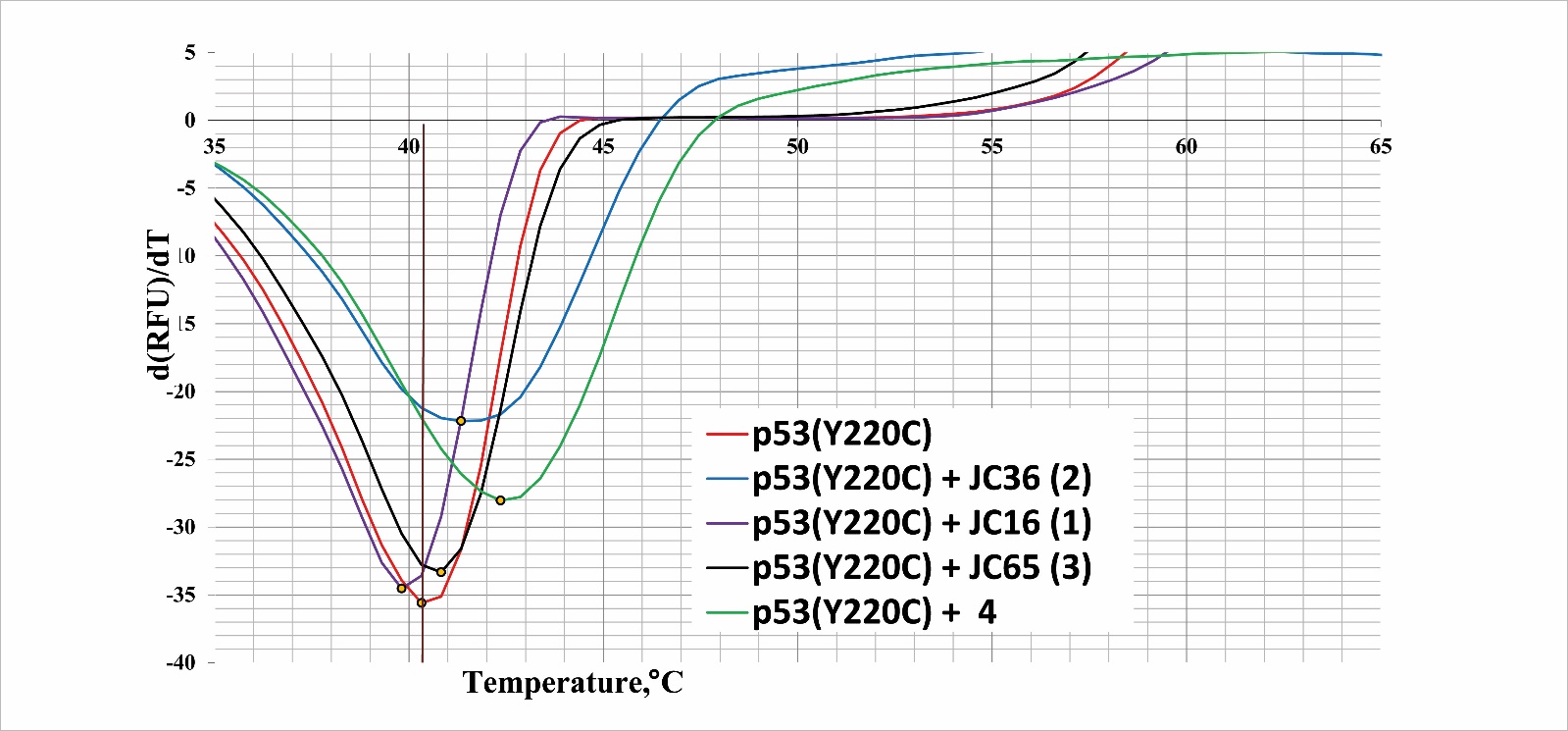


**Figure 1S.** **Thermal Stabilization of p53-Y220C DBD by Compounds Assessed via Differential Scanning Fluorimetry.** Derivative melting curves representing the fluorescence change as a function of temperature for p53-Y220C (10 μM) in the presence of compounds JC16 **(1)**, JC36 **(2)**, JC65 **(3)**, **4**, or DMSO control (500 μM each) were obtained using differential scanning fluorimetry. The curves corresponding to individual compounds are shown in distinct colors. Protein samples (10 μM) were mixed with Sypro Orange dye (10× final concentration) in 25 mM Hepes buffer (pH 7.2) containing 150 mM NaCl. The dark red vertical line indicates the melting temperature (Tm) of the p53-Y220C DBD as represented by the red curve (DMSO control). RFU denotes relative fluorescence units.


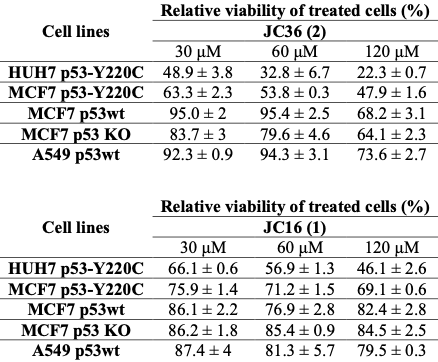


**Table 1S. Effect of Compounds on Cell Viability Across Different p53 Statuses Using MTS Assay.** Summary of relative cell viability for the most active compounds, JC16 **(1)** and JC36 **(2)**. Data are presented as mean ± SEM (n = 3).


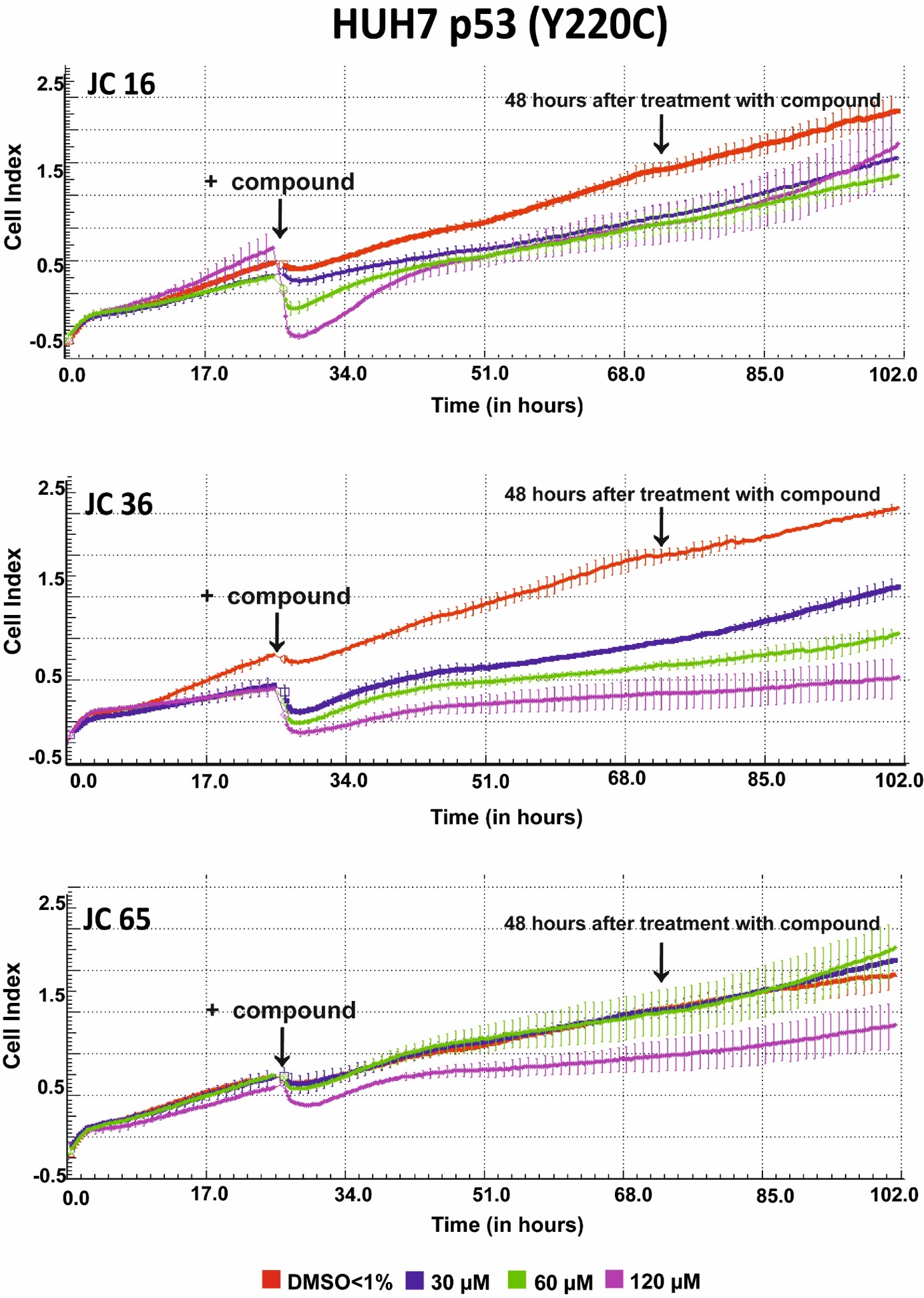


**Figure 2S.** **Real-Time Proliferative Activity of HUH7 p53-Y220C Cells Treated with JC16 (1), JC36 (2), and JC65 (3).** Proliferative activity of HUH7 p53-Y220C cells following treatment with compounds JC16 **(1)**, JC36 **(2)**, and JC65 **(3)** at concentrations of 30 µM, 60 µM, and 120 µM was assessed using the xCELLigence biosensor analyzer. Compounds were added 24 hours after cell seeding, and cell index values were monitored in real-time over a 102-hour incubation period. The graphs depict the cell index as a function of time, with measurements recorded for each concentration and compared to DMSO-treated controls (1% DMSO). Results are presented as mean ± SD of four technical replicates.


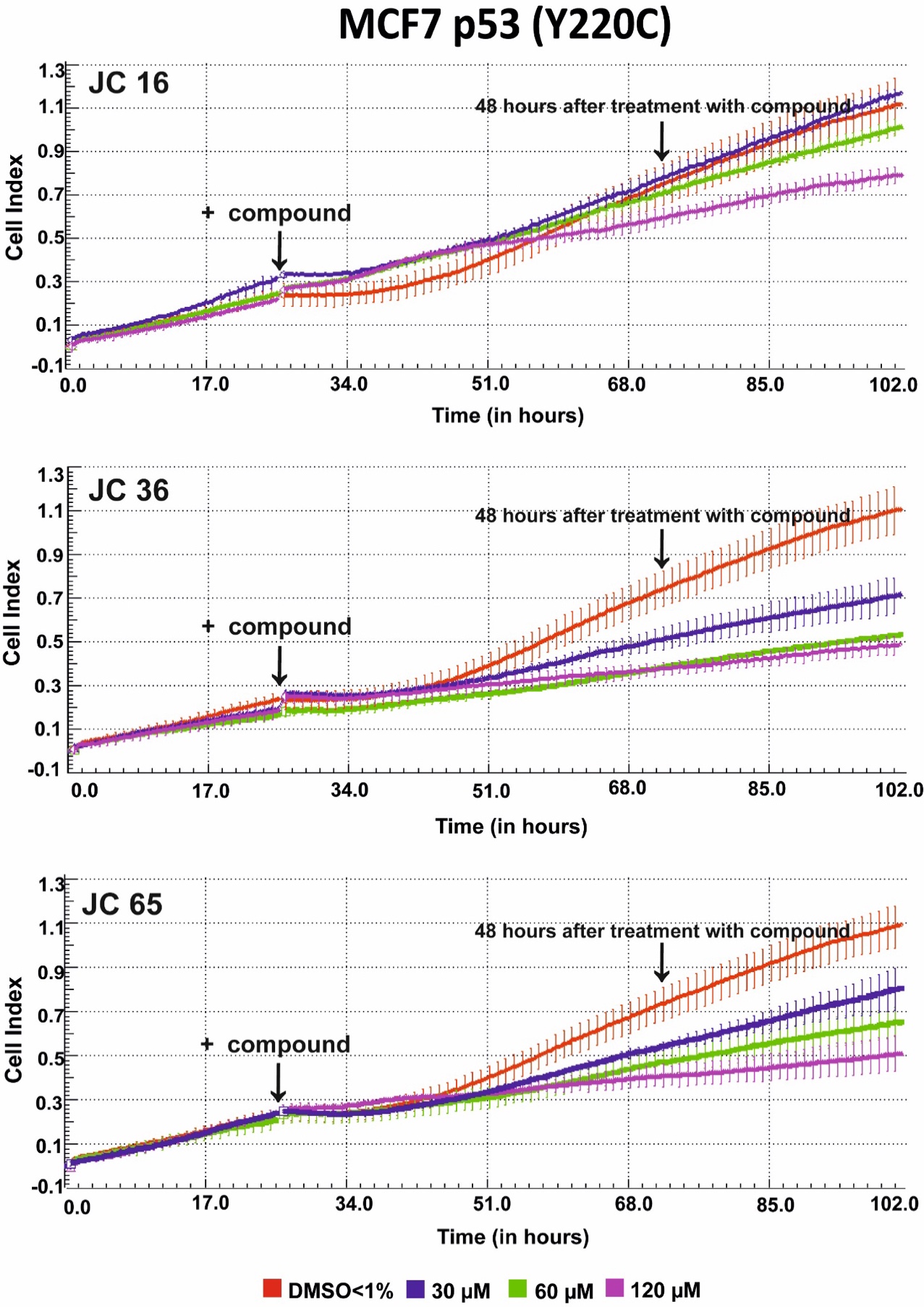


**Figure 3S. Real-Time Proliferative Activity of MCF7 p53-Y220C Cells Treated with JC16 (1), JC36 (2), and JC65 (3).** Proliferative activity of MCF7 p53-Y220C cells following treatment with compounds JC16 **(1)**, JC36 **(2)**, and JC65 **(3)** at concentrations of 30 µM, 60 µM, and 120 µM was analyzed using the xCELLigence biosensor cell analyzer. Compounds were added 24 hours after cell seeding, and cell index values were monitored in real-time over a 102-hour incubation period. Graphs depict the cell index as a function of incubation time for each compound and concentration, with comparisons to DMSO-treated controls (1% DMSO). Results are presented as mean ± SD of four technical replicates.


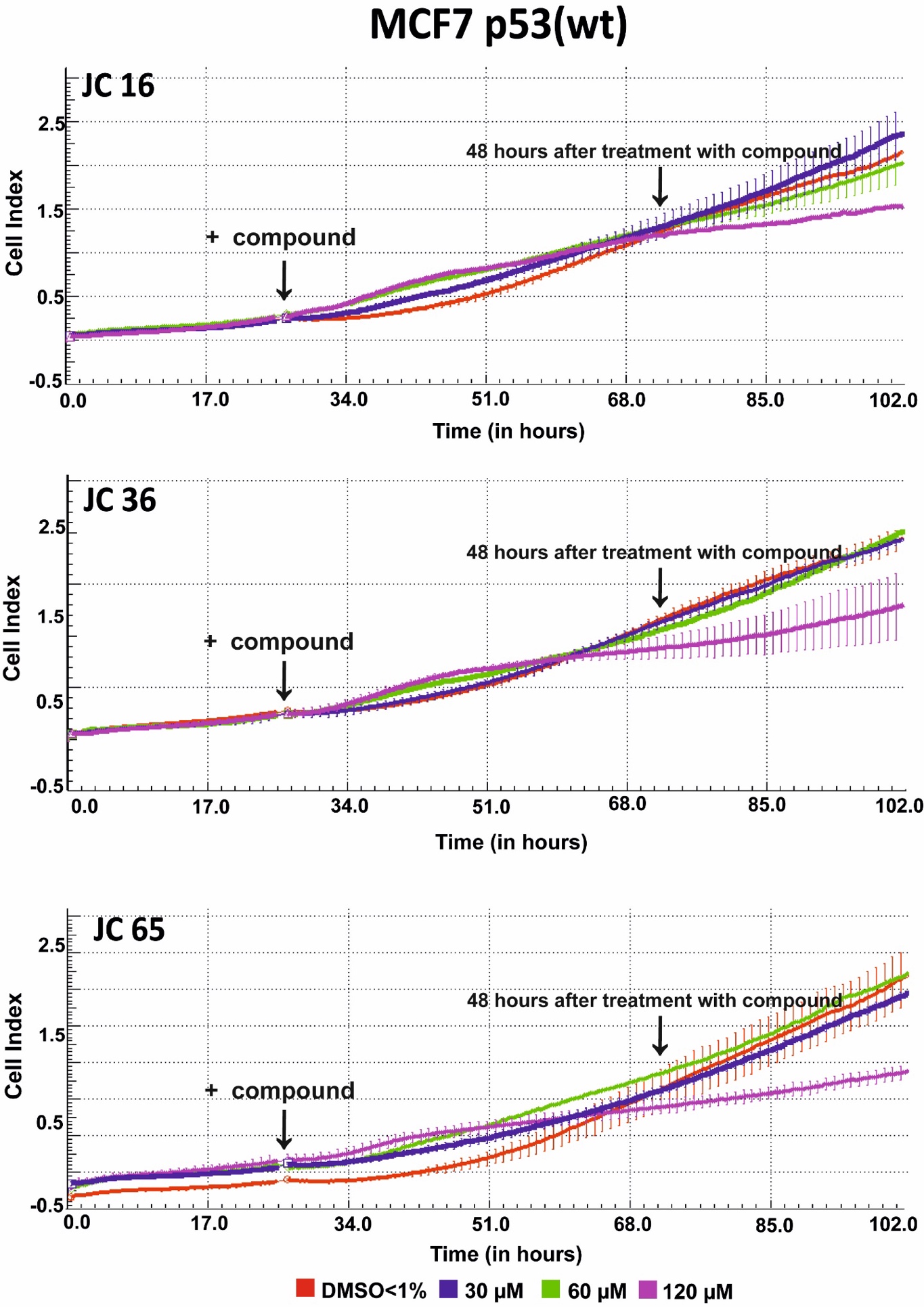


**Figure 4S**. **Real-Time Proliferative Activity of MCF7 p53wt Cells Treated with JC16 (1), JC36 (2), and JC65 (3).** Proliferative activity of MCF7 p53wt cells following treatment with compounds JC16 **(1)**, JC36 **(2)**, and JC65 **(3)** at concentrations of 30 µM, 60 µM, and 120 µM was analyzed using the xCELLigence biosensor cell analyzer. Compounds were added 24 hours after cell seeding, and cell index values were monitored in real-time over a 102-hour incubation period. Graphs illustrate the cell index as a function of incubation time for each compound and concentration, with comparisons to DMSO-treated controls (1% DMSO). Results are presented as mean ± SD of four technical replicates.


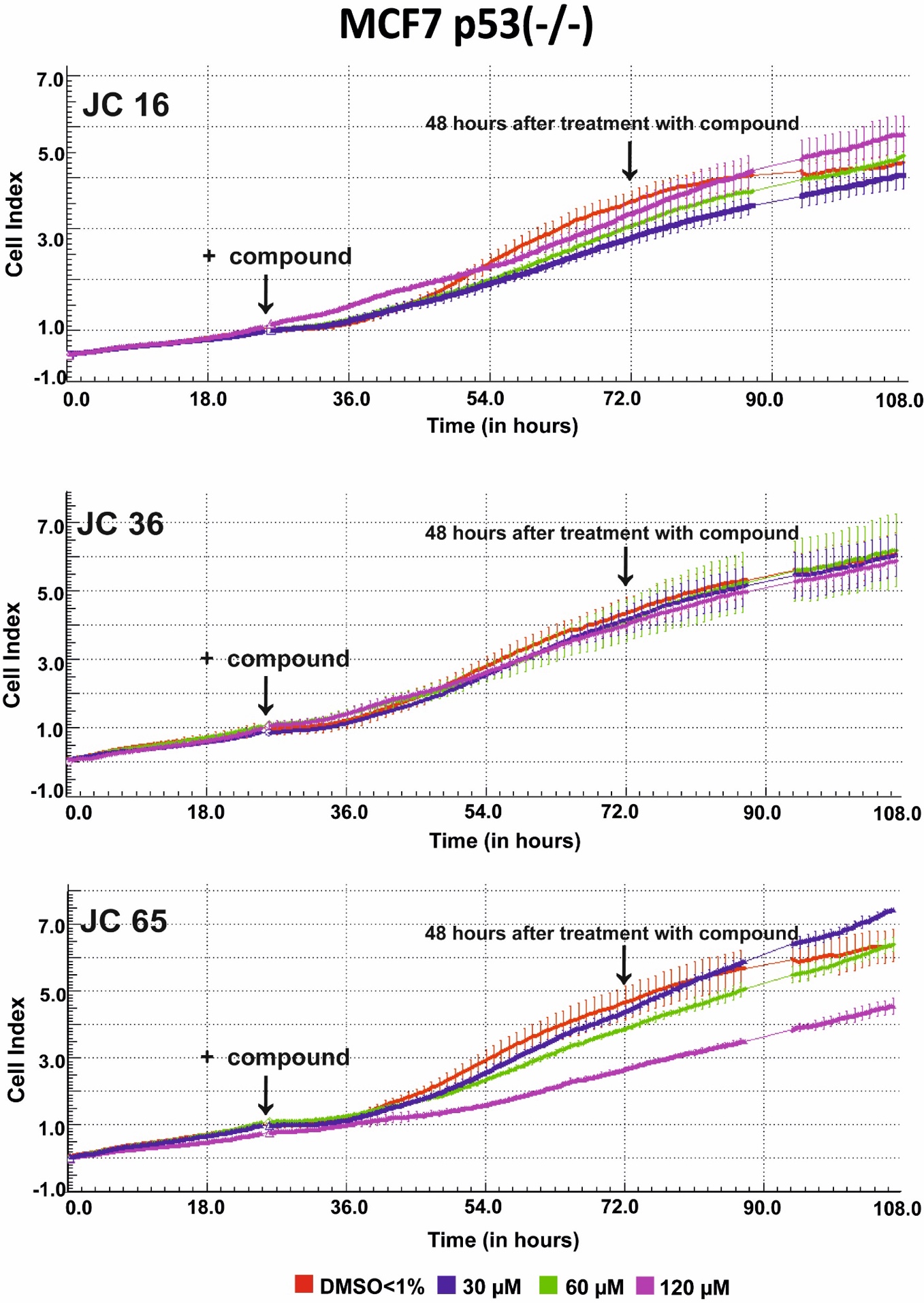


**Figure 5S. Real-Time Proliferative Activity of MCF7 p53 KO Cells Treated with JC16 (1), JC36 (2), and JC65 (3)**. Proliferative activity of MCF7 p53 KO cells following treatment with compounds JC16 **(1)**, JC36 **(2)**, and JC65 **(3)** at concentrations of 30 µM, 60 µM, and 120 µM was analyzed using the xCELLigence biosensor cell analyzer. Compounds were added 24 hours after cell seeding, and cell index values were monitored in real-time over a 108-hour incubation period. Graphs depict the cell index as a function of time for each compound and concentration, compared to DMSO-treated controls (1% DMSO). Results are presented as mean ± SD of four technical replicates.


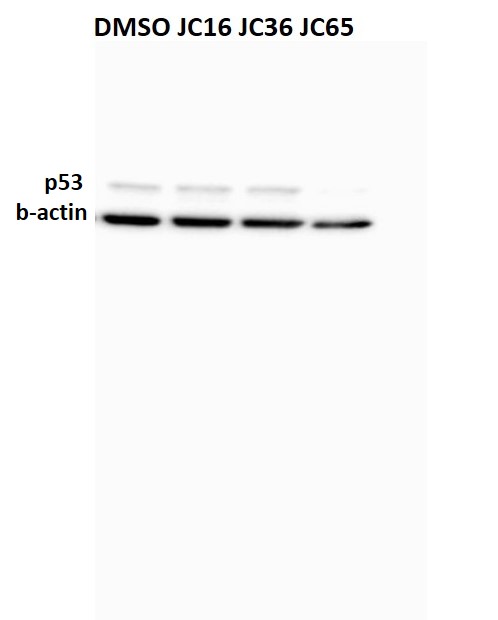

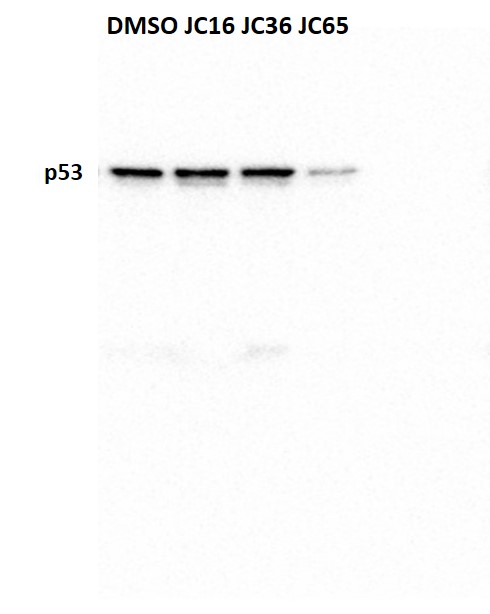


**Supplement for Figure 7. Effect of Compounds on the p53 Expression Levels in Cells with Different p53 Statuses.** Immunoblotting to determine the protein levels of p53 after JC16 (**1**), JC36 (**2**), and JC65(**3**) treatment for 24 h in MCF7 p53wt. Actin was used as a protein loading control. Left**:** shorter exposure time for p53; right: longer exposure time for p53


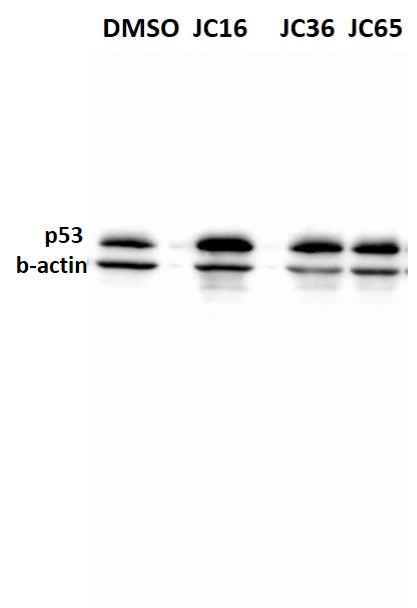


**Supplement for Figure 7. Effect of Compounds on the p53 Expression Levels in Cells with Different p53 Statuses.** Immunoblotting to determine the protein levels of p53 after JC16 (**1**), JC36 (**2**), and JC65(**3**) treatment for 24 h in MCF7 p53 Y220C. Actin was used as a protein loading control.

**
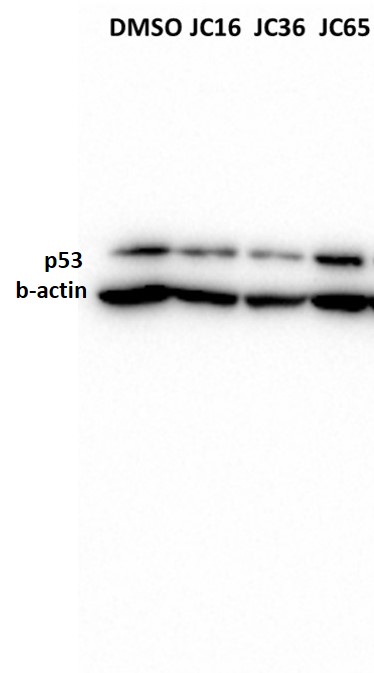
**

**Supplement for Figure 7. Effect of Compounds on the p53 Expression Levels in Cells with Different p53 Statuses.** Immunoblotting to determine the protein levels of p53 after JC16 (**1**), JC36 (**2**), and JC65(**3**) treatment for 24 h in HUH7 p53 Y220C. Actin was used as a protein loading control.


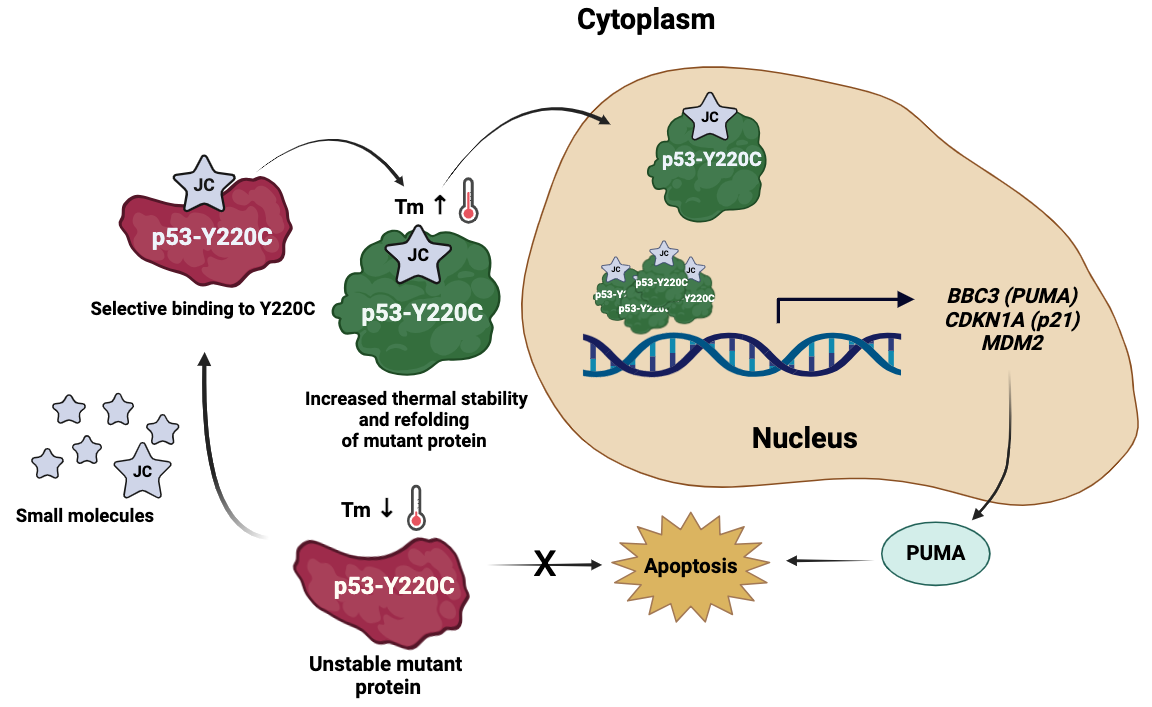


**Cover Image**
